# Prediction of the disease course in Friedreich ataxia

**DOI:** 10.1038/s41598-022-23666-z

**Published:** 2022-11-10

**Authors:** Christian Hohenfeld, Ulrich Terstiege, Imis Dogan, Paola Giunti, Michael H. Parkinson, Caterina Mariotti, Lorenzo Nanetti, Mario Fichera, Alexandra Durr, Claire Ewenczyk, Sylvia Boesch, Wolfgang Nachbauer, Thomas Klopstock, Claudia Stendel, Francisco Javier Rodríguez de Rivera Garrido, Ludger Schöls, Stefanie N. Hayer, Thomas Klockgether, Ilaria Giordano, Claire Didszun, Myriam Rai, Massimo Pandolfo, Holger Rauhut, Jörg B. Schulz, Kathrin Reetz

**Affiliations:** 1 Department of Neurology, RWTH Aachen University, 52074 Aachen, Germany; 2 JARA Brain Institute Molecular Neuroscience and Neuroimaging, Research Centre Jülich and RWTH Aachen University, 52056 Aachen, Germany; 3 Chair for Mathematics of Information Processing, RWTH Aachen University, 52062 Aachen, Germany; 4 Department of Clinical and Movement Neurosciences, Ataxia Centre, UCL-Queen Square Institute of Neurology, London, WC1N 3BG UK; 5 Unit of Medical Genetics and Neurogenetics, Fondazione IRCCS Istituto Neurologico Carlo Besta, 20133 Milan, Italy; 6 Sorbonne Université, Paris Brain Institute (ICM Institut du Cerveau), AP-HP, INSERM, CNRS, University Hospital Pitié-Salpêtrière, 75646 Paris, France; 7 Department of Neurology, Medical University Innsbruck, 6020 Innsbruck, Austria; 8 Department of Neurology, Friedrich Baur Institute, University Hospital, LMU, 80336 Munich, Germany; 9 German Center for Neurodegenerative Diseases (DZNE), 81377 Munich, Germany; 10 Munich Cluster for Systems Neurology (SyNergy), 81377 Munich, Germany; 11 Reference Unit of Hereditary Ataxias and Paraplegias, Department of Neurology, IdiPAZ, Hospital Universitario La Paz, 28046 Madrid, Spain; 12 Department of Neurology and Hertie-Institute for Clinical Brain Research, University of Tübingen, 72076 Tübingen, Germany; 13 German Center for Neurodegenerative Diseases (DZNE), 72076 Tübingen, Germany; 14 Department of Neurology, University Hospital of Bonn, 53127 Bonn, Germany; 15 German Center for Neurodegenerative Diseases (DZNE), 53127 Bonn, Germany; 16 Laboratory of Experimental Neurology, Université Libre de Bruxelles, 1070 Brussels, Belgium; 17 PhD Program in Neuroscience, School of Medicine and Surgery, University of Milano-Bicocca, 20126 Milan, Italy; 18 Department of Neurology and Neurosurgery, McGill University, Montreal, QC H3A 0G4 Canada

**Keywords:** Neurological disorders, Translational research

## Abstract

We explored whether disease severity of Friedreich ataxia can be predicted using data from clinical examinations. From the database of the European Friedreich Ataxia Consortium for Translational Studies (EFACTS) data from up to five examinations of 602 patients with genetically confirmed FRDA was included. Clinical instruments and important symptoms of FRDA were identified as targets for prediction, while variables such as genetics, age of disease onset and first symptom of the disease were used as predictors. We used modelling techniques including generalised linear models, support-vector-machines and decision trees. The scale for rating and assessment of ataxia (SARA) and the activities of daily living (ADL) could be predicted with predictive errors quantified by root-mean-squared-errors (RMSE) of 6.49 and 5.83, respectively. Also, we were able to achieve reasonable performance for loss of ambulation (ROC-AUC score of 0.83). However, predictions for the SCA functional assessment (SCAFI) and presence of cardiological symptoms were difficult. In conclusion, we demonstrate that some clinical features of FRDA can be predicted with reasonable error; being a first step towards future clinical applications of predictive modelling. In contrast, targets where predictions were difficult raise the question whether there are yet unknown variables driving the clinical phenotype of FRDA.

## Introduction

Friedreich ataxia (FRDA) is a rare autosomal-recessive, slowly progressing neurodegenerative spinocerebellar ataxia. While it is a rare disease with a prevalence of at most 1:20,000, it is at the same time the most common form of hereditary ataxia^1,2^.

The disease is caused by an expansion of GAA-repeats in the first intron of the *FXN* gene located on chromosome 9^3,4^. The gene encodes the Frataxin protein which is involved in iron-metabolism in mitochondria and is especially found in high metabolic cells such as in the muscles, nervous system and the heart^5^. In addition to homozygote GAA-repeat expansions in the *FXN* gene, there are heterozygote carriers of one GAA-repeat expansion and a pathogenic variant in *FXN* leading to a diverse clinical spectrum^6,7^.

The major clinical symptom is sensory and cerebellar ataxia. However, ataxia is usually accompanied by a wide array of non-ataxic symptoms such as scoliosis, pes cavus, cardiomyopathy, diabetes mellitus and urinary dysfunction^8^. The typical age at onset of the disease is during adolescence or childhood, with first symptoms often being imbalance, falls and/or scoliosis^2,8^. The heart disease seems to be independent from the neurological progression and is the leading cause of death in FRDA^9,10^. To this date, there is no cure or effective treatment for this devastating disease that usually leads to heavy impairment in both quality of life and independence for the affected individuals.

Previous work from EFACTS, the prospective registry by the European Friedreich Ataxia Consortium for Translational Studies (EFACTS; http://www.e-facts.eu)^11,12^ as well as studies from other groups^13–15^ have described the progression of the disease in FRDA over the course of several years. It was found that core symptoms related to spinocerebellar dysfunction become gradually worse over time and clinical instruments for measuring ataxia such as the scale for rating and assessment of ataxia (SARA) also indicate worsening with each subsequent examination, despite high variability in scores. A core challenge regarding predictions and modelling of the disease course of FRDA is that the disease is progressing rather slowly and additionally the complex clinical picture varies to a significant extent beyond the defining symptoms.

To enable answering legitimate questions patients affected by FRDA might have about the disease progression and to also potentially enable informed estimates about an individual’s disease progression for clinical use, we exploratively attempted to model and predict disease severity by employing techniques of statistical learning. Having at least an informed estimate on how to answer such a question could help in individualising medicine for individuals with this disease and in the long-term such data could support planning the interval of examinations and also help patients and their care takers making preparations for potential future disabilities and limitations affecting daily life. Furthermore, an informed estimate could help in situations where data is lost or when visits are only possible in a limited manner. As the modelling approach was explorative we did refrain from formulating hypotheses.

## Methods

### Subjects

All patients included in this analysis participated in the annual examinations for the EFACTS longitudinal database and had genetically confirmed FRDA. Retrieved data included only patients for which monitoring had been completed up to the fifth annual visit (fourth follow-up), leading to 602 patients with a total of 2306 data points. All subjects or their authorised surrogates gave informed consent before participation and all procedures were reviewed and approved by the institutional ethics committee of the medical faculty of RWTH Aachen University (reference number: EK 057/10) and carried out in accordance with the Declaration of Helsinki^16^.

### Modelling implementations

Data analysis and modelling were carried out using the R programming language version 4.0^17^ and the Python programming language version 3.7 (https://python.org). Most important libraries used for modelling were the tidymodels framework^18^ in R as well as scikit-learn^19^ in Python.

### Variables of interest

We identified potential variables of interest (targets) from the data. The main goal was to select items characterising overall disease severity and progression in an individual as well as items containing information about symptoms that are especially debilitating for an affected individual. These included clinical instruments characterising current disease severity; namely the SARA total score^20^, activities of daily living (ADL) total score^21^, and spinocerebellar ataxia (SCA) functional assessment (SCAFI) sub-scale results (i.e. 8-metre-walk-test mean seconds, nine-hole-peg-test mean seconds, PATA task mean syllables)^22^. Furthermore, presence of cardiological symptoms (arrhythmia, hypertrophy, left-ventricular hypertrophy, repolarisation abnormalities) was a group of targets of interest. We included loss of ambulation in two stages based on the spinocerebellar degeneration functional score^23^ and an additional dichotomous item coding wheelchair-boundness. Full loss of ambulation or more severe was defined as being wheelchair-bound or more severely affected, while near loss of ambulation was defined as being able to walk with support of two sticks or more severely affected.

As cardiac symptoms are an important non-ataxia feature in FRDA, we created an additional variable specifying the presence of any cardiological symptom. For creating this variable, we used all dichotomous items in the regular (annual) EFACTS examination coding the presence of cardiac symptoms. If any of these items indicated pathological findings, the created variable was set to 1, otherwise it was set to 0.

Furthermore, we were interested in cardiac hypertrophy. However, as data quality regarding the presence of cardiac hypertrophy was in parts insufficient for modelling due to missing data and information based on external clinical reports, we created an additional variable *hypertrophy risk* coding the likelihood of presence of cardiac hypertrophy on a scale from zero (most likely no hypertrophy) to five (very likely hypertrophy), based on these items giving the presence of hypertrophy and left-ventricular-hypertrophy, septum-thickness and a free-form text item for cardiac diagnoses which was evaluated programmatically using regular expressions. If one of the input data points was missing, the risk item was set to be missing as well; however if at some time points input data was available and missing at other visits, the available data was re-used for the time points where it was missing. In case that value was 2 or higher, we considered hypertrophy to be likely present in a patient, and for modelling the variable was then dichotomised into unlikely hypertrophy ($<2$) and likely hypertrophy ($\ge$ 2).

For modelling the selected targets, relevant predictors (clinical and genetic routine features) were selected from the data. A critical point here was to ensure no feature leakage (including features that implicitly contain information about the target) would be present in the modelling, as many of the variables available in the EFACTS database measure disease progression in some way. By correlating features with targets and selecting variables based on topic knowledge we identified a *core set* of features, namely age at disease onset, age at examination, GAA-repeats on both *FXN* alleles separately, gender, first symptom of the disease and presence of problems during the neonatal phase. To examine the predictive performance reachable with more limited predictor sets we also used a minimal predictor set containing only age of disease onset and disease duration (below referred to as the *minimal set*) and another set extending the minimal set with GAA-repeats on the shorter allele (below referred to as the *small set*).

### Predictive models

For all types of targets, we compared the predictive performance of various suitable model types against each other and also against a trivial predictor (see below) establishing a baseline of predictive performance.

#### Data processing

For modelling we split the data into a training set and a test set, where 80% of available data was used for the training set and the remaining data for the test set. To ensure that inclusion of a single subject in both sets would not artificially inflate model performance, we generally split the data so that all time points from a single subject would only be included in one of the split data sets, but not in both. However, for evaluating the impact of subject effects in the data we additionally modelled ADL and SARA scores with this constraint removed.

A set of processing steps was applied to the data. Processing was done on the training set initially and afterwards applied to the test set based on what was learned for the training data. Predictors were removed if at least 10% of the data was missing; in a similar fashion subjects were removed if 10% or more of data points for a subject were missing from the selected predictors. Then, categorical data was dummy coded and the remaining missing values were imputed using a k-nearest-neighbour classifier using Gower’s distance and *k* set to 5. Imputation affected 19 values. Next, predictors with zero or near-zero variance were removed, in this case affecting one predictor. Predictors were then centred and scaled to be in [0, 1]. A check for highly correlated predictors was done (with $|r| \ge .85$), but this did not pertain to any of the included predictors.

The removal of subjects with missing predictor data during processing resulted in a training set with 1030 observations, while the test set had 276 observations, leaving 368 unique subjects with multiple time points in the training set and 93 in the test set. Missing data in targets was ignored in this step and only removed when fitting models.

#### Continuous targets

For continuous targets models were evaluated based on the root mean squared error (RMSE) of the model’s predictions against the actual values. RMSE characterises the difference between prediction and actual observation (truth), but emphasises larger errors more than smaller errors. We also provide the mean absolute error (MAE), which has the same goal as RMSE, but treats all data points equally. The smaller both values are, the better is the prediction. The trivial predictor employed here was using the mean of the training data as *prediction* for all observations in the test data. Both RMSE and MAE reflect the predictive error in the scale of the target, thus these values have to be interpreted within the target’s context and con not be compared between targets in a straightforward manner.

Modelling families that were employed were Linear Regression, Lasso Regression^24^, Random Forests^25^ and Gradient Tree Boosting (XGB^26^).

#### Categorical targets

For categorical targets models were evaluated using the receiver operating characteristic - area under the curve (ROC-AUC) based on probabilities assigned to class predictions compared to the actual values. ROC-AUC characterises a binary model’s quality and is 0 if all predictions are the opposite from the truth, a random predictor would be expected to reach a value of 0.5 and a perfect prediction is a value of 1. The trivial predictor for categorical targets was using the most common category from the training data as *prediction* for all observations in the test data. For comparing modelling performance against the trivial predictor accuracy was used, as calculating ROC-AUC scores on the trivial predictor is not sensible.

Modelling families were largely similar to what was used for continuous targets and were: Logistic Regression (Generalised Linear Model), Logistic Lasso Regression, Support Vector Machines^27^, Random Forests and Gradient Tree Boosting (XGB).

#### Model fitting

Where available we tuned some hyperparameters of models using a grid search and 10-fold-cross-validation, for finding values leading to optimal predictive performance.

The hyperparameters we tuned were the regularisation parameter *C* for logistic regression and its variant Lasso regression (inverse of regularisation strength $\lambda$), the regularisation parameter *C* and Gaussian kernel coefficient $\gamma$ for SVM, the number of trees for random forests and the loss regularisation, learning rate and depth for XGB.

#### Variable importance

Where available, we calculated estimates of variable importance for all predictors that were entered into the models. For generalised linear models coefficients were used, characterising the weight of each predictor. As predictors were scaled and centred in [0, 1] this allows for a crude estimation of the influence of the predictors compared to each other. For tree-based models Gini importance was used, characterising the mean decrease in impurity from a predictor. In less technical terms the importance associated with a predictor can be thought of as a metric of the improvement of the prediction when creating tree splits on that variable. Finally, for SVM permutation importance was used, characterising the importance of a predictor for the model by randomly shuffling predictors and evaluating how much this corruption affects the outcome. To help with comparisons, absolute values of importance scores were re-scaled to [0, 1], with the least influential predictor set to 0 and the most influential predictor set to 1.

## Results

### Sample characteristics

A detailed description of the sample at baseline is available elsewhere^8,11,12^. The average length of GAA-repeats on the shorter allele was 591.0 (standard deviation [SD] = 269.2, range: [6, 1200]), with 573 patients being homozygotes for the GAA-repeat expansion. Average disease duration at baseline was 18.2 years (SD = 10.2, range: [1, 55]), with the mean age at onset being 15.5 years (SD = 10.4, range: [1, 65]). Using a cut-off of age at onset of 25 or higher, the sample included 99 patients of late-onset FRDA. At baseline the mean SARA score was 22.0 (SD = 9.6, range: [1.5, 40]), while the mean ADL score was 14.6 (SD = 7.8, range: [0, 35]). As for the SCAFI, the average time to complete the 8-metre-walk task was 12.2 s (SD = 13.8, range: [3.5, 127.5]), while for the 9-hole-peg-test subjects took an average of 67.0 s (SD = 44.0, range: [15.4, 275]) to complete the task. For the PATA task the mean number of syllables was 19.2 (SD = 6.0, range: [2.5, 39]).

For the categorical variables of interest, at baseline, cardiac hypertrophy was present in 216 patients with FRDA (35.8% of the sample), arrhythmia in 20 (3.3%), left ventricular hypertrophy in 66 (11.0%) and repolarisation abnormalities in 253 (42.0%). The cardiac hypertrophy risk feature indicated likely cardiac hypertrophy in 162 patients with FRDA (26.9%), while 462 of the 602 patients at baseline (76.7%) had at least one cardiac symptom. At baseline, 291 patients with FRDA (48.3%) were affected by full loss of ambulation, while 390 (64.8%) were characterised as near loss of ambulation or more severe. The distributions of categorical items in the training set is given in Table 1, also providing a picture of how these variables are distributed among the patients in this study.

**Table 1:** Distribution of categorical targets in the training set.

Variable	Present	Absent	Missing
Full LoA	679 (65.9%)	351 (34.1%)	0 (0%)
Near LoA	826 (80.2%)	204 (19.8%)	0 (0%)
Arrhythmia	46 (4.5%)	625 (60.7%)	359 (34.9%)
Hypertrophy	396 (38.4%)	528 (51.3%)	106 (10.3%)
L Ventric Hy	123 (11.9%)	506 (49.1%)	401 (38.9%)
Repol Abn	415 (40.3%)	242 (23.5%)	373 (36.2%)
Hyper risk	489 (47.5%)	344 (33.4%)	197 (19.1%)
Any Cardio	696 (67.6%)	206 (20.0%)	128 (12.4%)

The table shows presence, absence and missing data of categorical features in the training set, illustrating eventual class imbalances. *LoA* loss of ambulation; *L Ventric Hy* left ventricular hypertrophy; *Repol Abn* repolarisation abnormalities; *Hyper Risk* engineered variable coding the likelihood of the presence of cardiac hypertrophy.

### Prediction of continuous targets

Predictions for the SARA using the full predictor set reached RMSE between 6.49 and 6.75, with the trivial predictor achieving a RMSE of 9.07 points. Using the minimal set RMSE ranged between 7.04 and 7.18 points, while with the small set values between 6.64 and 7.16 were reached. For predicting the scores at the next annual visit only, predictive performance was largely in a similar range.

**Figure 1:**
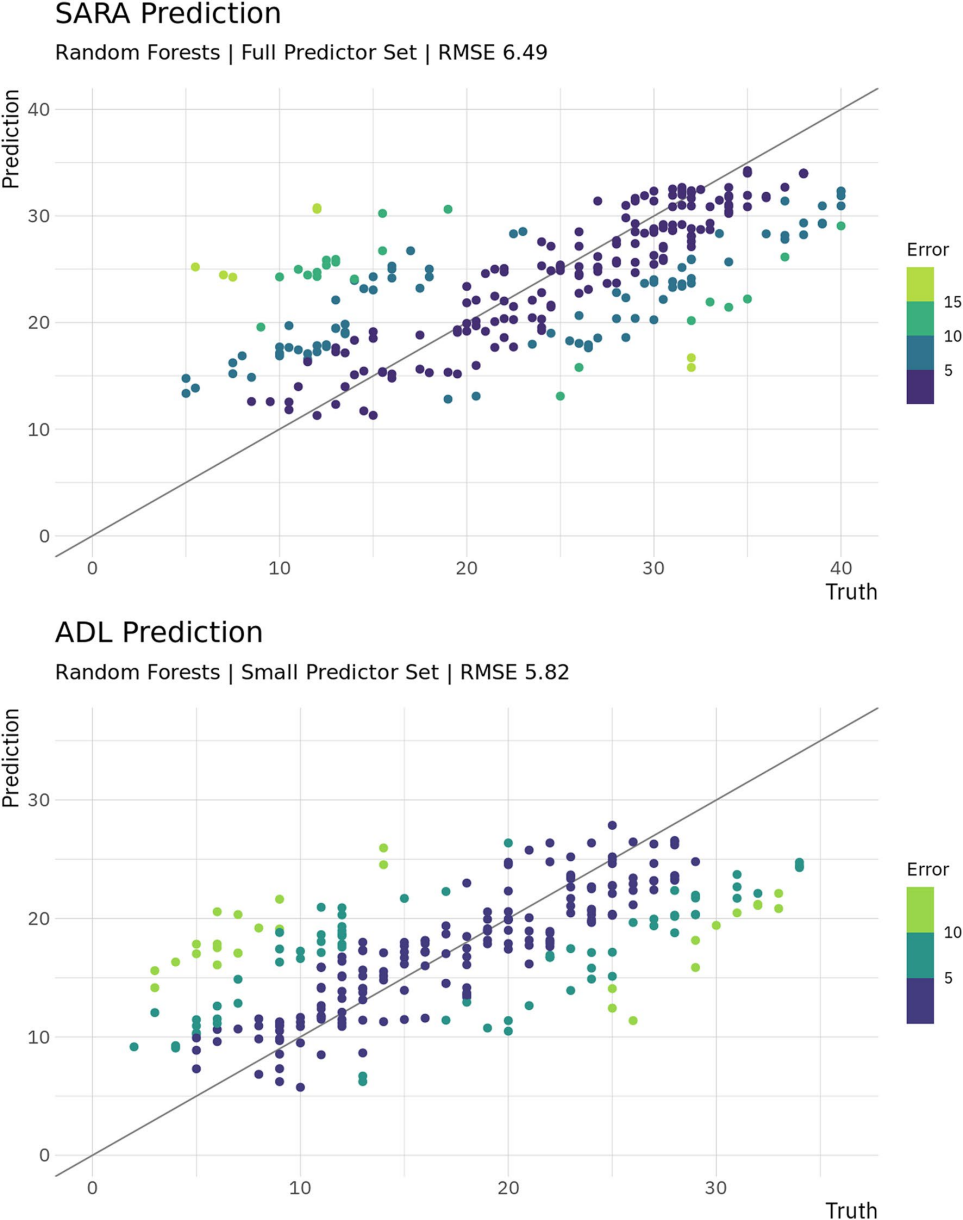
Truth and prediction for SARA and ADL. Shown are the predictions plotted against actual values for the best performing model for both clinical instruments. A perfect prediction would have all values on the diagonal line.

**Figure 2:**
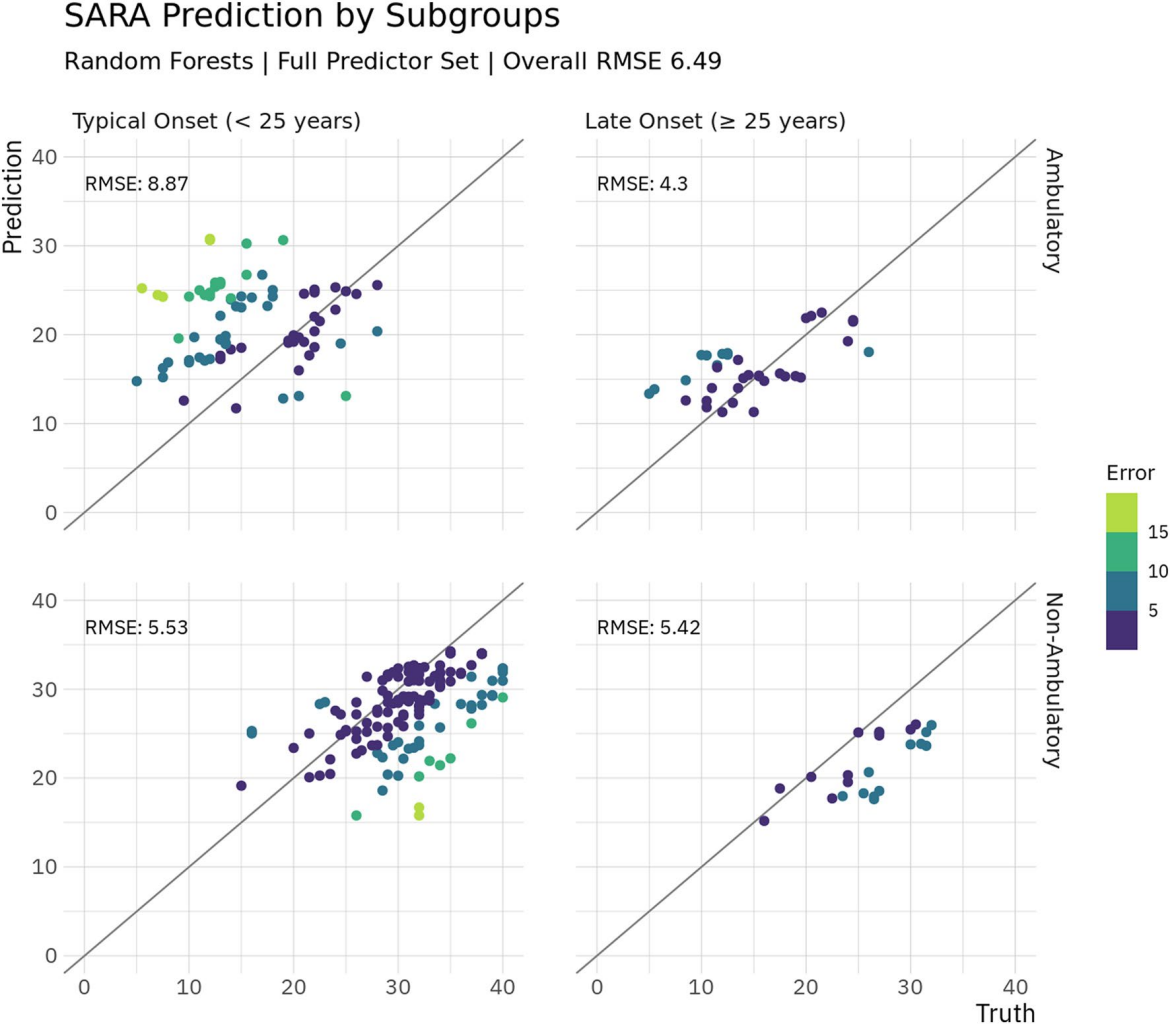
SARA Prediction by Subgroup. Predictions are shown grouped by age of onset and ambulation, where non-ambulatory is defined as being permanently wheelchair bound. For each section of the grid the RMSE in the subgroup is given. Perfect predictions would have all values on the diagonal lines.

For the ADL predictions reached a RMSE between 5.77 and 6.58 points, while the trivial predictor reached 7.85 points RMSE. For the minimal set, we found similar performance with RMSE being between 6.14 and 6.20 points, with the small set reaching RMSE between 5.82 and 6.16. Similar as for the SARA, predictions of scores at the next visit only were of similar quality. Predictions for the SARA and ADL are visualised in Fig. 1. Additionally, there is visualisation of SARA predictions by onset and ambulation in Fig. 2, illustrating that predictions were the worst for ambulatory patients with disease onset before 25 years old (RMSE of 8.87), while for typical onset non-ambulatory patients as well as late-onset patients, errors were considerably smaller (RMSE 4.30–5.42).

As for the SCAFI predictions of performance in the 8-metre-walk-test reached RMSE between 10.13 and 12.73 s across all predictor sets, with the trivial predictor being included in this range at 11.00 s. For the nine-hole-peg-test predictions reached RMSE from 38.03 to 48.39 s across all predictor set, with this range also including the trivial predictor at 46.36 s. Finally, for the PATA task RMSE ranged between 5.23 and 7.66 syllables across all predictor set; a range which also included the trivial predictor reaching a RMSE of 6.13.

For details on all models for continuous data, including those models with the constraint of subjects being exclusive to either training set or test set removed, see Table 2.

**Table 2:** Predictions of continuous data.

Target	Trivial RMSE	Pred Set	Best Fam	Best RMSE	Best MAE	Norm RMSE
SARA	9.07	Full	RF	6.49	5.07	0.19
SARA + 1	8.99	Full	RF	6.53	5.07	0.19
SARA S	8.63	Full	RF	3.81	2.82	0.10
ADL	7.85	Small	RF	5.82	4.63	0.18
ADL + 1	7.80	Small	RF	5.71	4.59	0.19
ADL S	7.48	Full	RF	3.55	2.62	0.11
SCAFI w8m	11.00	Full	Lasso	10.13	7.07	0.13
SCAFI w8m + 1	8.35	Full	RF	7.37	5.69	0.19
SCAFI 9hpt	46.36	Full	RF	38.03	27.23	0.18
SCAFI 9hpt + 1	47.21	Small	RF	37.16	27.58	0.16
SCAFI PATA	6.13	Full	LinReg	5.23	4.13	0.17
SCAFI PATA + 1	6.19	Full	LinReg	5.31	4.17	0.17

The table shows the best performing models for each continuous target. Targets marked with + 1 are the results at the next visit only, while targets marked with *S* indicate modelling with allowing subjects to be present in training and test set at the same time. The best RMSE is the overall best result of all models, the best MAE is the MAE achieved from that model, thus not necessarily the overall best MAE reached. The trivial predictor for continuous targets was *predicting* the mean for all data points. For normalising RMSE, values were divided by the range of data. *RMSE* root mean squared error; *Pred Set* predictor set; *Best Fam* model family reaching the best result; *MAE* mean absolute error; *Norm* Normalised; *RF* random forest; *LinReg* inear regression; *w8m* 8 m walk test; *9hpt* nine hole peg test.

### Prediction of categorical targets

For the presence of cardiac hypertrophy as encoded in a binary item, ROC-AUC scores across all predictor sets varied between 0.55 and 0.69 with the accuracy at the highest ROC-AUC score being 0.69, while the trivial classifier reached an accuracy of 0.57. For hypertrophy at the next visit ROC-AUC scores were in the range 0.59 and 0.75, the best model here achieving an accuracy of 0.65, compared to a trivial baseline of 0.54.

For repolarisation abnormalities ROC-AUC scores ranged from 0.52 to 0.68, the model with the highest ROC-AUC score reaching an accuracy of 0.65, while the trivial classifier reached an accuracy of 0.52. For the prediction of repolarisation abnormalities at the next visit, ROC-AUC scores were between 0.55 and 0.73; the best model having an accuracy of 0.71, while the trivial predictor reached 0.52.

Some approaches turned out to reach performances not better or even worse than the trivial predictor, this was true for prediction of arrhythmia as well as left ventricular hypertrophy.

For the *presence of any cardiological symptom*, we reached ROC-AUC values in the range from 0.50 to 0.75; the best model reaching an accuracy of 0.71, being only slightly better than the trivial classifier with an accuracy of 0.68.

For risk of hypertrophy the range of ROC-AUC scores of evaluated models ranged between 0.58 and 0.67. Here, the best model reached an accuracy of 0.61, being only marginally better than the accuracy of 0.59 in the trivial baseline.

As for full loss of ambulation ROC-AUC scores where between 0.68 and 0.84, the best model having an accuracy of 0.74 compared to the trivial classifier with 0.59. Regarding predictions for full loss of ambulation at the next visit, performance was largely similar with the model’s ROC-AUC score being 0.85, achieving an accuracy of 0.73, while the baseline was an accuracy of 0.60.

Finally, for near loss of ambulation we were able to achieve ROC-AUC scores between 0.66 and 0.82. The accuracy that was reached here was 0.79, compared to 0.77 of the trivial prediction. Performance in predicting near loss of ambulation at the next visit was worse with the best ROC-AUC score being 0.78 and reaching an accuracy of 0.81, compared to 0.77 for the trivial predictor.

**Figure 3:**
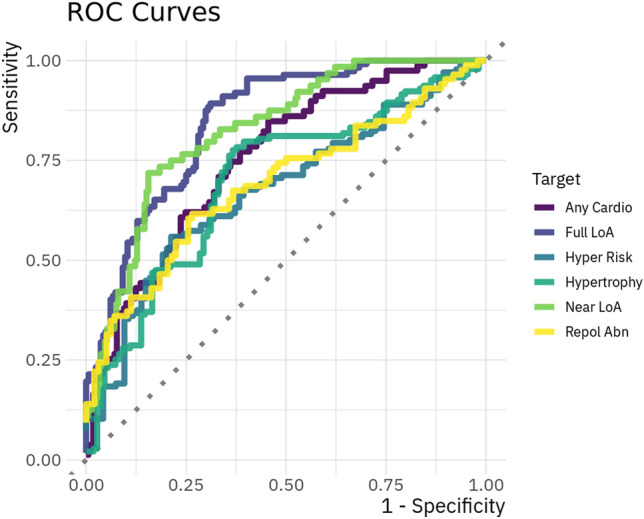
ROC curves for selected classification models. Shown are the ROC curves of six classifiers. Abbreviations: LoA - Loss of Ambulation; Repol Abn - repolarisation abnormalities.

For all details on models for categorical targets see Table 3; in addition ROC curves for selected models are visualised in Fig. 3.

**Table 3:** Predictions of categorical data.

Target	Trivial Acc	Pred set	Best Fam	Best RA	RA Acc	Best Acc
Full LoA	0.59	Full	XGB	0.83	0.73	0.77
Full LoA + 1	0.60	Full	XGB	0.85	0.73	0.77
Near LoA	0.77	Small	LogReg	0.82	0.79	0.83
Near LoA + 1	0.77	Minimal	RBF SVM	0.78	0.81	0.81
Arrhythmia	0.92	Minimal	RBF SVM	0.69	0.92	0.94
Arrhythmia + 1	0.89	Minimal	RBF SVM	0.66	0.89	0.90
Hypertrophy	0.57	Minimal	LogReg	0.69	0.69	0.69
Hypertrophy + 1	0.54	Minimal	XGB	0.75	0.65	0.70
L Ventric Hy	0.86	Small	RF	0.64	0.89	0.86
L Ventric Hy + 1	0.84	Minimal	XGB	0.71	0.85	0.85
Repol Abn	0.53	Small	LogReg	0.68	0.65	0.65
Repol Abn + 1	0.52	Small	LogReg	0.73	0.71	0.71
Hyper Risk	0.59	Minimal	Lasso	0.67	0.61	0.65
Hyper Risk + 1	0.58	Minimal	LogReg	0.73	0.65	0.67
Any Cardio	0.68	Minimal	RF	0.75	0.73	0.73
Any Cardio + 1	0.68	Minimal	RF	0.78	0.72	0.75

The table shows the best performing models for each categorical target. Targets marked with + 1 are the results for the next visit only. The best ROC-AUC score is the overall best result of all models, the RA accuracy is the accuracy achieved from that model, thus not necessarily the overall best accuracy reached. The best overall accuracy is given in the best accuracy column. The trivial predictor for categorical targets was *predicting* the most common category for all data points. *Acc* Accuracy; *RA * ROC-AUC score; *Pred Set* predictor set; *Best Fam* model family reaching the best result; *XGB* extreme gradient boosting; *RBF SVM* support vector machines with radial basis function; *RF* random forest; *LogReg* logistic regression; *LoA* Loss of Ambulation; *L Ventric Hy* left ventricular hypertrophy; *Repol Abn* repolarisation abnormalities; *Hyper Risk* engineered variable coding the likelihood of the presence of cardiac hypertrophy.

### Variable importance

**Figure 4:**
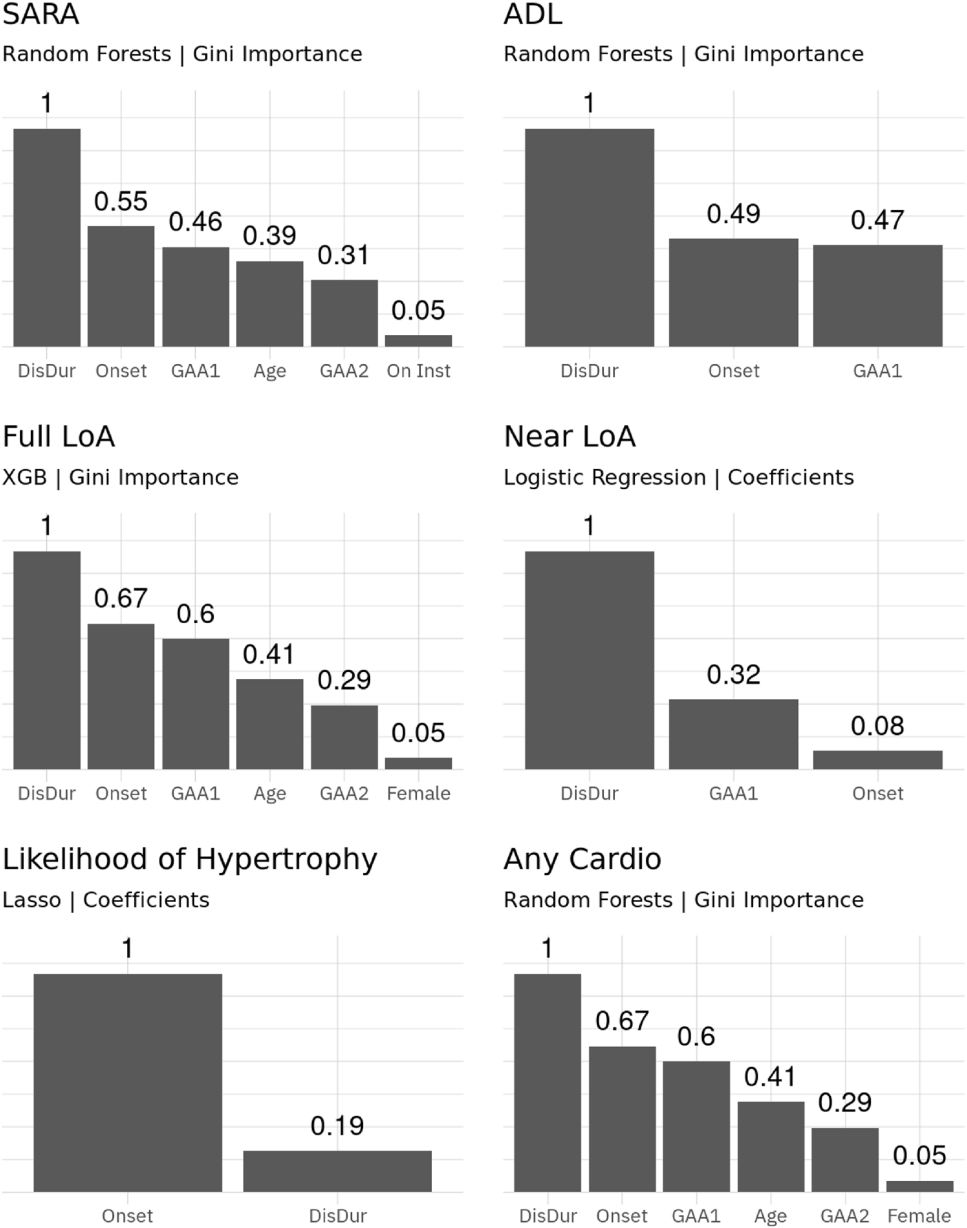
Variable importance for selected models. Shown are the up to 6 most important features for the best models. Scores are scaled as fraction of highest value. Abbreviations: DisDur - disease duration; Onset - age of disease onset; Female - patient is female.

We briefly assessed variable importance. Generally, independent of the model disease duration was usually the most influential variable. Variables such as GAA-repeats, age at onset and age were the next most important variables, with all other included predictors only being of small importance. A visualisation of variable importance for selected models is presented in Fig. 4.

## Discussion

In this work, we used techniques of statistical learning to model state and severity of disease in FRDA based on a large sample. To the best of our knowledge, this is the first work using a purely predictive approach in FRDA with the greater aim of enabling individualised medicine and care in the long-term. For some measures of disease severity modelling worked reasonably well, such as for the SARA and ADL clinical scales and loss of ambulation. However, modelling performance was rather insufficient when it came to the SCAFI subtests as well as most targets on cardiac symptoms.

Both the SARA and the ADL are well-established important clinical instruments to assess severity and progression of the disease for which relations between *a priori* measures and outcome are well known^1,8,11^. They were among the outcomes that we could model with the least amount of error, fitting well in the relation between a priori measures and clinical outcome. Previous works have suggested that SARA scores increase at a rate of about one point per year of disease duration^11^. However, recently it was reported that the progression is not constant and faster at the beginning of the disease course^12,28^, while it was also reported that the ADL might be more sensitive than the SARA at various disease stages^12^.

There has been some focus on loss of ambulation in FRDA, which is a symptom that tremendously affects patients’ well-being^1^. A recent work has estimated the time to loss of ambulation^29^. In addition to a clear relation between age of disease onset as well as disease duration and loss of ambulation, a relatively clear progression of lost functions could be shown leading up to eventual loss of ambulation. Furthermore, a recent paper on the EFACTS data has shown that clinical instruments show differential progression rates over time before and after loss of ambulation^12^. We did include two distinct stages of the progression towards loss of ambulation. Predictive performance was relatively good especially for full loss of ambulation, reinforcing the notion that there is a clear relation between eventual loss of ambulation and the data we used as predictors here, like genetics, age at onset, disease duration and first symptom of the disease. Furthermore, we did explore whether predictions of SARA differed by ambulation and onset and found that for non-ambulatory patients of all onset ages predictions worked better than for ambulatory typical onset patients in particular, but the amount of data per group is not too large and thus should be interpreted with caution. Also, it should be pointed out that recently it has been suggested that progression in the SARA is not equally driven across its items, with items relating to the status of ambulation being more sensitive than others in still ambulatory patients^12^. This in turn suggests that there is less room for variability in patients that are already non-ambulatory. Details like these should be addressed in subsequent more focused works, potentially enabling better predictive performance.

Predictions of SCAFI subtests did turn out to only have a poor performance in FRDA^30^. This is unexpected as progressive ataxia as found in FRDA should reflect in a clinical test focused on the motor system. One issue to raise with the SCAFI in FRDA is that the 8-metre-walk test cannot be completed by a large number of patients due the eventual loss of ambulation associated with the disease^1,31^. As patients, unable to perform the test, were not included in the modelling, this should not have affected the outcome too much. However, this did lead to a starkly reduced sample size for this subtest. As for the 9-hole peg test, previous works have shown that performance deteriorates with time^12^, also with the CCFS^32^ but the error in prediction was rather large here. This suggests that on the one hand performance in this task becomes progressively worse, on the other hand the performance itself is mostly unrelated to a priori markers. Assuming the SCAFI is a valid instrument to assess disease severity in FRDA, the quality of prediction here might point towards yet unknown variables driving the clinical state as measured by SCAFI subtests; but this has to remain speculative as there is no direct evidence for it.

A similar outcome as for the SCAFI was found for items coding the presence of cardiological symptoms. The situation is different here than for the SCAFI for several reasons, though. First of all, for many of the symptoms queried in the annual EFACTS examinations the presence of symptoms is often very skewed in the way that symptoms are either very common or very rare without many changes over the observed amount of time. This is for example true for arrhythmia and left ventricular hypertrophy, where the class imbalance is extremely large. We did not account for this in our modelling approaches, thus impairing the predictive quality reached. Further, research points towards the onset and progression of this symptom group not being fully understood yet in the context of FRDA^33^. One study found that only a subset of patients in FRDA show progressive decline in cardiac function, but the features that distinguish both groups of patients are unknown^10^. Without reliable separation of these phenotypes, predicting cardiac symptoms in FRDA will of course be rather difficult.

While based on objective measurements^34^, there is some amount of error introduced by clinicians^35^ when diagnosing cardiac hypertrophy. We attempted to counteract this by combining several sources of information on whether cardiac hypertrophy is present into a new variable, but we did not reach better predictive performance on that new target representing *likelihood of cardiac hypertrophy* compared to other cardiological targets. Additionally, when trying to predict whether any cardiac symptom is present, predictions were of similar quality as when trying to predict individual cardiac symptoms. This suggests that there is no common overreaching relationship between the predictors included here and the manifestation of heart disease in FRDA. This fits well in the overall uncertainty surrounding cardiac symptoms in FRDA.

Especially for the modelling of clinical measurements it should be pointed out that some amount of variation might be expected. A recent trial for a remote examination of the SARA found that scores could vary up to a similar extent as the prediction error we found here over a short amount of time^36^. While the trial investigated a remote examination and non-FRDA ataxias, it nonetheless supports the idea that a score of a clinical examination is not as static as it might appear. In contrast, research seeking to quantify retest-reliability of the modified Friedreich Ataxia Rating Scale as well as the SARA in patients of FRDA did find rather high retest-reliability^37,38^. Despite standardised operating procedures effects due to multiple and changing examiners as well as shifts in subjective criteria where examinations depend on a clinician’s rating are concerns over the long-term and ways to address these issues should be sought. Stability of results from rating scales is both a challenge and a limitation when applying techniques of statistical learning to clinical instruments and at the moment there is not much data to draw conclusions on how FRDA and the instruments commonly used here are affected by this issue.

Numerous more predictors were included in the present work compared to a priori information included in previous works. With a wider range of available predictors there might be potential for improved predictive accuracy if the used predictors convey additional information. However, the data presented here suggests that in some cases rather small predictor sets led to a superior predictive performance and even if the full predictor set led to the best performance, the difference to the smaller ones was usually rather minor. Given previous literature it is not unsurprising that especially the number of GAA-repeats, disease duration and age of disease onset together led to a very reasonable predictive performance.

For a small set of targets we did remove the constraint of subjects being exclusive to either training or test set and obtained a much better predictive performance. This is not too surprising, but this does suggest that what the models’ trained on in that scenario was mainly the individual combination of predictors. Obviously, it is near-impossible to gather all information that might have influenced the course of the disease over a person’s life. But this does point towards further, yet unknown, variables having substantial influence on an individual’s disease course. The uncertainty around cardiological symptoms that is established in the literature^10,33^ and the results obtained in the present work for the SCAFI support the idea that there are important, but yet unknown, variables influencing the disease course in FRDA.

The predictive performance that was found here should of course be put into context, looking at what can be achieved in other diseases. Using techniques from the domain of machine learning to enable individualised medicine is still a relatively new approach. In FRDA one work used a recommendation algorithm to fill missing items in the SARA scale^39^. Similar approaches used methods of supervised statistical learning to identify core signs for disease progression^40^ or used a quantitative motor task to identify variables relevant to disease progression^41^. Our work differs insofar from previous studies, as that we here attempt a broad prediction of the clinical state in FRDA. In non-FRDA ataxias similar approaches for identification of important features^42,43^ as well as classification between healthy controls and patients have been carried out^44^, reaching an accuracy of 0.88. Considering machine learning approaches beyond ataxia, in Parkinson’s disease, studies making use of deep learning reached accuracies between 0.8 and 0.85 on UPDRS data using voice recordings^45^ and motor data as inputs^46^. Similarly, in Alzheimer’s disease prediction of disease severity scores could be demonstrated with an accuracy of 0.83 based on a variety of clinical and biological measures^47^. A study using deep learning to predict clinical dementia rating of patients at future clinical examinations reached an accuracy of 0.99, but the model was tested against data of subjects it was trained on^48^. Overall, this suggests that at least some of the results presented here are not too far away regarding predictive performance to what is currently available in the literature. It should be noted that we here undertook a *one-size-fits-all* approach, applying a wide range of techniques to many targets without specifically optimising our models. For eventual routine use of predictions in a clinical setting as well as follow-up works, much more optimised approaches focusing on a very small number of targets are needed.

In the longer-term this work can be viewed as a first step towards individualised medicine^49^ in FRDA. The idea here is that a set of core variables allowing for prediction of the future disease course within reasonable margin of error can augment clinicians’ work for better planning of routine examinations as well as pointing out patients at risk for certain symptoms. Of course, at the moment the predictive performance we report here is not good enough for clinical use and open questions remain, but we demonstrate the general feasibility of such approaches in a rare disease like FRDA.

The inclusion of measures characterising the state of neurodegeneration as well as quantitative motor data as predictors would certainly be an interesting follow-up work, although modelling approaches are likely to become more complex with more diverse features. Despite being to some degree sensitive to the applied (pre-) processing, measures characterising the central nervous system are suffering less from issues regarding objectivity than clinical scales. Characteristic alterations of the spinal cord, brain stem and cerebellum in FRDA have recently been shown^50,51^ and thus seem like a viable variable to include in predictive modelling of the disease course.

### Limitations

There are limitations in the work presented here. First, while in the context of EFACTS large amounts of data are regularly gathered in a standardised manner, missing data was the main drawback in this work. Especially when researching a rare disease, it is much better to have a smaller, but complete dataset, than having a wide range of variables, but large amounts of missing data. However, we are aware that examinations in the context of the study can be strenuous and for severely affected patients there are constraints regarding how long of an examination they can participate in and also which kinds of examinations are practicable. We are grateful for the ongoing participation of many individuals affected by this disease.

Second, we did not employ additional techniques like resampling strategies or introducing synthetic data where class imbalance was a problem, leading to likely worse predictive performance than what could be theoretically possible.

Finally, it should be noted that on the other hand predictive modelling and statistical learning in a clinical context bear a large potential for improving decision making and enabling individualised medicine, on the other hand they should not replace attention and care by medical professionals.

## Conclusions

In conclusion, in this paper we demonstrated that certain clinical instruments and features relevant for FRDA can be predicted to an extent that allows for an informed estimate about a patient’s disease severity. However, parts of the clinical phenotype of FRDA are still not well understood and this is reflected in subpar predictive performance when attempting to establish predictive modelling for these clinical features. This work can be seen as a promising first step towards a more individualised medicine in FRDA. Much more work, both on optimising modelling for the most promising targets, as well as on the understanding of FRDA as a whole is necessary.

## Data Availability

The datasets generated during and/or analysed during the current study are not publicly available for protecting patient privacy but are available from the corresponding author on reasonable request.
